# New Acorane-Type Sesquiterpene from *Acorus* *calamus* L.

**DOI:** 10.3390/molecules22040529

**Published:** 2017-03-26

**Authors:** Juan Li, Jianping Zhao, Wei Wang, Lin Li, Lan Zhang, Xiao-Fang Zhao, Qing-Ru Liu, Fang Liu, Min Yang, Ikhlas A. Khan, Shun-Xiang Li

**Affiliations:** 1 Hunan Province Engineering Research Center of Bioactive Substance Discovery of TCM, Hunan University of Chinese Medicine, Changsha 410208, Hunan, China; forever_lijuan@163.com (J.L.); wangwei402@hotmail.com (W.W.); woshizxiaof@sina.com (X.-F.Z.); lqr199106@hotmail.com (Q.-R.L.); fliu0825@126.com (F.L.); 2 National Center for Natural Products Research, University of Mississippi, University MS 38677, USA; zjianping@hotmail.com (J.Z.); ikhan@olemiss.edu (I.A.K.); 3 Key Laboratory of Neurodegenerative Diseases of Ministry of Education, Capital Medical University, Beijing 100053, China; linli97@hotmail.com (L.L.); lanizhg@hotmail.com (L.Z.); 4 Shanghai Institute of Materia Medica, Chinese Academy of Sciences, Shanghai 201203, China; ymn77@163.com

**Keywords:** *Acorus calamus* L., Araceae, acorane-type sesquiterpene, biosynthetic pathway, cell proliferation

## Abstract

A new sesquiterpene, named neo-acorane A (**1**), and two known ones, acoric acid (**2**) and calamusin D (**3**), were isolated from a 95% ethanol extract of the rhizome parts of *Acorus calamus* L. Their structures were elucidated by spectroscopic methods, and the absolute configurations were determined by single-crystal X-ray diffraction analysis. Compounds **1** and **2** are nonisoprenoid sesquiterpenoids, likely biosynthesized from an acorane-type sesquiterpene by oxidative fission of the six- or five-membered ring. Moreover, compounds **1** (10 μM), **2** (5 μM and 10 μM) and **3** (10 μM) showed cell proliferation activity on the SK-N-BE (2) cell line.

## 1. Introduction

*Acorus calamus* L. is a member of the monocot family Araceae and is widely distributed among the northern temperate and subtropical regions of Asia, North America, and Europe. In China, the rhizomes of *A. calamus* have long been used as a folk medicine for treatments of phlegm syncope, stroke, epilepsy, palpitation, amnesia, tinnitus, deafness, dyspepsia-inducing abdominal pain, dysentery, rheumatic pain, eczema and scabies [1]. Previous phytochemical studies on *A. calamus* discovered the presence of sesquiterpenes, alkaloids, flavones, and fatty acids [2,3,4,5,6]. Bioactivity studies showed that extracts and compounds from the plant had anti-dementia, anti-microbial, anti-epileptic, insecticidal, and anti-diabetic activities [2,3,4,5,6]. These facts encouraged us to reinvestigate and find new and bioactive secondary metabolites, which can treat neurodegenerative diseases such as Alzheimer’s disease, epilepsy, and stroke. In our continuous search, we had isolated and elucidated a new sesquiterpene, neo-acorane A (**1**), and two known ones from the ethanol extract of the rhizomes of *A. calamus*. Details of the structural elucidation and plausible biogenesis, and the cell proliferation activity of compounds **1**–**3** are reported herein.

## 2. Results and Discussion

### 2.1. Phytochemical Study

**Figure 1:**
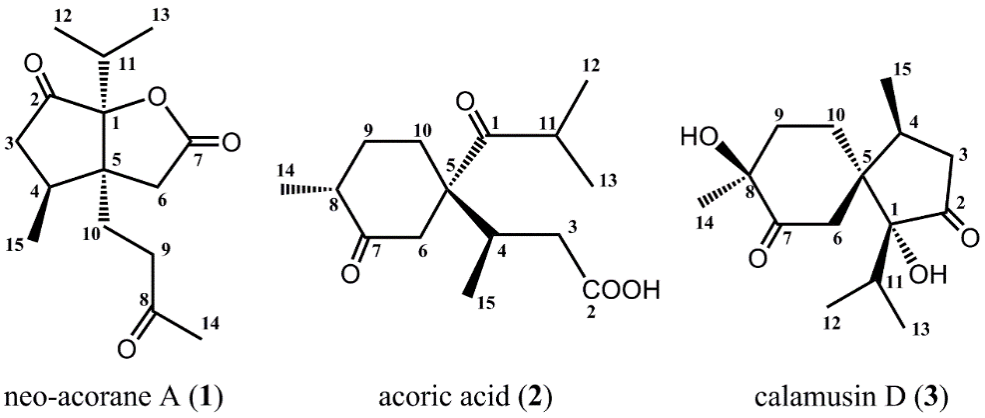
Structures of isolated compounds from *A. calamus*.

As part of our search for novel medicinal compounds from plants, we investigated the rhizome parts of *A. calamus*. Chromatographic separation and purification resulted in the isolation and full identification of a new sesquiterpene, neo-acorane A (**1**), and two known ones, acoric acid (**2**) and calamusin D (**3**) (Figure 1).

Neo-acorane A (**1**) was obtained as colorless needle crystals (MeOH) and was determined to have a molecular formula of C_15_H_22_O_4_ on the basis of the HRESIMS data at *m*/*z* 267.1643 [M + H]^+^ (calcd. 267.1596), 289.1455 [M + Na]^+^ (calcd. 289.1416). The IR spectrum indicated the characteristic absorption bands of carbonyl groups (1778, 1749, 1715 cm^−1^). From the analysis of the ^1^H- and ^13^C-NMR spectra (Table 1) of compound **1**, together with the examination of the HSQC and HMBC experiments, 15 carbon signals of the ^13^C-NMR data were identified as four methyls, four methylenes, two methines, and five quaternary carbons (assigned as three carbonyl groups, and two sp^3^ hybridize). It can be speculated that compound **1** is a sesquiterpene lactone with the characteristic signals, such as one downfield-shifted signal of the carbonyl group of lactone (δ_C_ 173.8, C-7), and one oxygenated quaternary carbon (δ_C_ 94.8, C-1).

**Figure 2:**
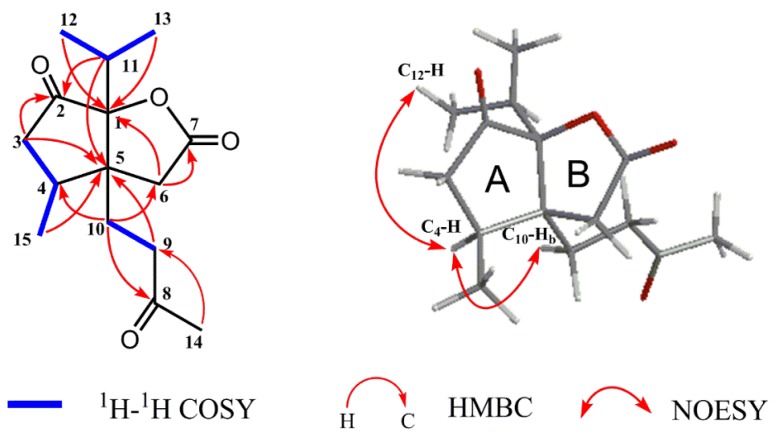
Key ^1^H-^1^H COSY, HMBC, and NOESY correlations of compound **1**.

The planar structure of compound **1** was constructed by detailed analyses of ^1^H-^1^H COSY, HSQC, and HMBC spectra. Three proton-bearing structural fragments, corresponding to H_2_-3/H-4/H_3_-15, H_2_-9/H_2_-10, and H_3_-12/H-11/H_3_-13, were observed by analysis of the ^1^H-^1^H COSY spectra (Figure 2). The HMBC spectrum of compound **1** exhibited the correlations of H_2_-3/C-2 and C-5, of H_3_-15/C-5, of H_2_-6/C-1 and C-7, of H_2_-9/C-5, of H_2_-10/ C-4, C-6, and C-8, of H-11/ C-2, and C-5, of H_3_-12/C-1, of H_3_-13/C-1, of H_3_-14/C-8, and C-9. Through analysis of one ketone carbon (*δ*_C_ 213.2, C-2) and the key HMBC correlations, as depicted with arrows from H to C (Figure 2, Table 1), it was confirmed that unit A was a cyclopentanone moiety and linked with unit B via the bridge carbons C-1 and C-5.

**Figure 3:**
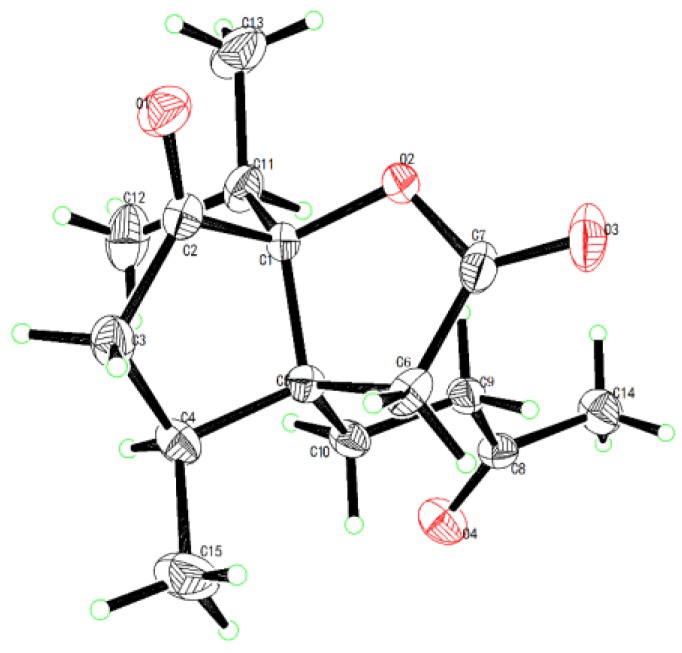
X-ray crystallographic structure of compound **1**.

The relative configuration of compound **1** was determined by NOESY correlations (Figure 2). The key NOE correlations of H-4/H-10b and H-4/H_3_-12 indicated they are on the same face and were arbitrarily assigned as *α*-oriented. The structure of compound **1** was further confirmed by X-ray crystallographic analysis using Cu K*α* radiation, and its absolute configuration was unambiguously determined as 1*S*, 4*S*, 5*S* (Figure 3). The chemical name of compound **1** is (3a*S*, 4*S*, 6a*S*)-3a-(3-oxobutyl)-4-methyl-6a-isopropyl-tetrahydro-2*H*-cyclopenta [b] furan-2,6(3*H*)-dione [7,8], and we named it neo-acorane A. NMR, HRESIMS, IR and UV spectra of compound **1** are available in Supplementary Materials.

**Figure 4:**
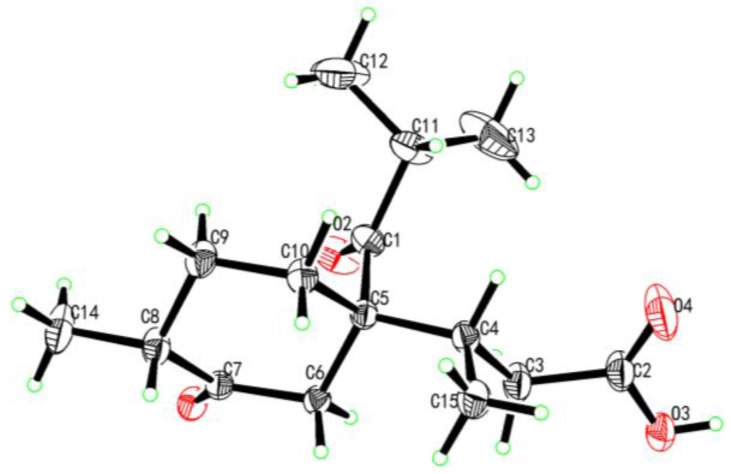
X-ray crystallographic structure of compound **2**.

The structures of compounds **2** and **3** were identified by comparison of their physiochemical properties and spectrometric data with those reported in the literature [2,3]. The absolute configuration of compound **2** was previously unknown, and it was determined as 4*S*, 5*S*, 8*R* by X-ray crystallographic analysis using Cu K*α* radiation (Figure 4).

### 2.2. Possible Biosynthetic Pathway

**Scheme 1:**
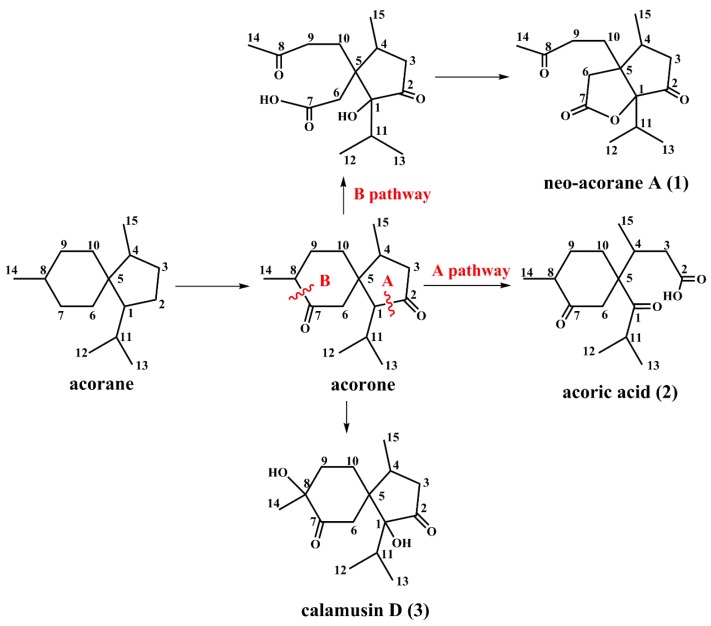
Possible biosynthetic pathways of compounds **1**–**3**.

So far, many sesquiterpenes from *A. calamus* mainly consist of acorane, guaiane, cadinane, and elemane-type sesquiterpenes [9,10,11]. Compounds **1** and **2** are nonisoprenoid sesquiterpenoids, and may derive from the acorane skeleton with new biogenetic pathways. We speculated that compound **2** should be formed via the oxidative fission of the single bond between C-1 and C-2 (the A pathway) [3], and compound **1** might be biosynthesized by the oxidative fission of the single bond between C-7 and C-8 firstly, and then forms a five-membered lactone ring through dehydration and cyclization (the B pathway) (Scheme 1). Thus compounds **1** and **2** represent two new skeleton types deriving from acorane-type sesquiterpene.

### 2.3. Cell Proliferation Assay

**Figure 5:**
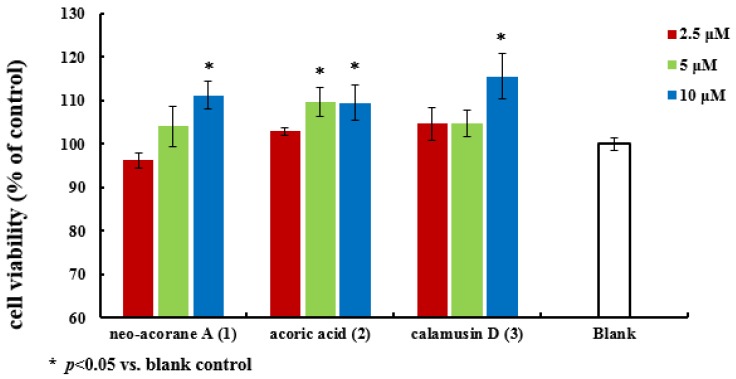
Cell proliferation activity of compounds **1**–**3**.

All of the isolated compounds (**1**–**3**) were evaluated for cell proliferation activity on the SK-N-BE (2) cell line with 1‰ dimethylsulfoxide (DMSO) medium as a blank control. The results (Figure 5) showed that compounds **1** (10 μM), **2** (5 μM and 10 μM) and **3** (10 μM) significantly promoted cell proliferation activity comparing with the blank control in vitro.

## 3. Materials and Methods

### 3.1. General Experimental Procedures

Melting points were measured with a X-4 melting point apparatus (Beijing KWF Sci-Tech Development Co., Ltd., Bejing, China). Specific rotations were measured with a Rudolph Research AutoPol IV polarimeter (Rudolph Research Flangers, Hackettstown, NJ, USA) at room temperature. UV spectra were collected by a Hewlett-Packard 8452A UV-vis spectrometer (Hewlett Packard, Palo Alto, CA, USA). IR spectra were measured with a Bruker Tensor 27 and MIRacle ATR FT-IR spectrometers (Bruker Optics, Fremont, CA, USA). NMR spectra were recorded on an Agilent DD2-500 NMR spectrometer ( Agilent, Santa Clara, CA, USA) with a OneNMR probe at 500 MHz for ^1^H and 125 MHz for ^13^C using the pulse programs provided in the Agilent Vnmrj 3.2 software. All chemical shifts were quoted on the *δ* scale in ppm using residual solvent as the internal standard (CDCl_3_: 7.26 ppm for ^1^H-NMR, 77.2 ppm for ^13^C-NMR). Coupling constants (*J*) are reported in Hz. HRESIMS were measured on HR-ESI-TOF-MS spectrometer (Agilent Technologies, Palo Alto, CA, USA) with the AnalystTM QS software (Agilent Series 1100 SL) for data acquisition and processing. X-ray single-crystal diffraction experiment was carried out on a Bruker APEX-II CCD detector (Bruker, Fremont, CA, USA ) employing graphite monochromated Cu K*α* radiation. Column chromatograph (CC) was performed on silica gel (100–200 and 200–300 mesh; Qingdao Marine Chemical Factory, Qingdao, China) and ODS (YMC-gel, 60–80 µm, YMC Co. Ltd, Kyoto, Japan). Precoated silica gel GF_254_ plates (Qingdao Marine Chemical Factory, Qingdao, China) were used for TLC. Spots were visualized using UV light (254 and/or 365 nm) and by spraying with 5% (*v*/*v*) H_2_SO_4_ ethanol solution followed by heating at 105 °C.

### 3.2. Plant Material

*A. calamus* was collected in December of 2011 at Dawei Mountain, Liuyang City, Hunan Province, China, and authenticated by Prof. Ta-Si Liu (School of Pharmacy, Hunan University of Chinese Medicine, Changsha, China). A voucher specimen (No. 20111211) was deposited in Hunan Province Engineering Research Center of Bioactive Substance Discovery of TCM at Hunan University of Chinese Medicine, Changsha, China.

### 3.3. Extraction and Isolation

The air-dried rhizomes of *A. calamus* (10 kg) were powdered and extracted with ethanol (95% *v*/*v*) three times at room temperature for 24 h each time. The combined extraction was filtered and concentrated under reduced pressure to give a residue (1.1 kg, oily residue), which was then suspended in water and partitioned with petroleum ether (PE, 60–90 °C), trichloromethane (CHCl_3_), ethyl acetate (EtOAc) and *n*-butanol (*n*-BuOH) in succession, to give PE (618.6 g), CHCl_3_ (58.9 g, oily residue), EtOAc (5.2 g) and *n*-BuOH (16.0 g) extracts, respectively. The CHCl_3_ extract was subjected to dry column on silica gel (200–300 mesh, 6.0 cm× 100 cm, 1.0 kg; PE-acetone, 50:1 to 0:50) to afford six main fractions (Frs.1–6). Fr. 2 (10.5 g) was chromatographed on a silica gel column (200–300 mesh, 4.0 × 60 cm, 300 g, PE-acetone, 30:1 to 0:30) to obtain eight subfractions (Frs. 2.1–2.8). Fr. 2.4 (1.0 g) was further purified by preparative HPLC using MeOH‑H_2_O (70:30, 2 mL/min) as the eluent to afford compounds **3** (5.0 mg, t_R_ = 25 min) and **1** (6.0 mg, t_R_ = 38 min). Fr. 2.6 (1.2 g) was purified by recrystallization to obtain compound **2** (300 mg).

### 3.4. Spectroscopic Data of New Compound

*Neo-acorane A* (**1**): Colorless crystals (MeOH) in needle-type; m.p. 91–92 °C; ${\left[\alpha \right]}_{D}^{20}$ +21.3° (*c* 1.87 × 10^−3^, MeOH); UV (MeOH) *λ*_max_ (log *ε*): 203 (2.69) nm; IR (KBr) ν_max_ 2968, 2936, 1778, 1749, 1715, 1227, 1186, 1167, 1012, 958, 895 cm^−1^; NMR data see Table 1; (+)-HRESIMS *m*/*z* 267.1643 [M + H]^+^ (calcd. for C_15_H_23_O_4,_ 267.1596), 289.1455 [M + Na]^+^ (calcd. for C_15_H_22_O_4_Na, 289.1416).

### 3.5. Crystallographic Data of Compounds

Data collection was performed with a Bruker APEX-II CCD detector employing graphite monochromated Cu K*α* radiation with Bruker SAINT software. The programs used to solve and refine structures were SHELXS-97 and SHELXL-97, respectively.

Crystal data for neo-acorane A (**1**): C_15_H_22_O_4_ (M = 266.33 g/mol), orthorhombic, space group P 21 21 21, a = 7.88520(10) Å, α = 90°, b = 12.4542(2) Å, β = 90°, c = 15.1058(2) Å, γ = 90°, V = 1483.45(4) Å^3^, Z = 4, T = 296(2)K, λ(CuKα) = 1.54178 Å, Dcalc = 1.130 g/cm^3^, F(000) = 544, independent reflections 2677 [R(int) = 0.1239], 9.203° < 2θ < 130.2°, the final R1 was 0.0429 (I > 2σ(I)) and wR2 was 0.1173 (all data).

Crystal data for acoric acid (**2**): C_15_H_24_O_4_ (M = 268.34 g/mol), orthorhombic, space group P 21 21 21, a = 9.4392(4) Å, α = 90°, b = 11.4671(4) Å, β = 90°, c = 14.3042(5) Å, γ = 90°, V = 1548.29(10) Å^3^, Z = 4, T = 296(2) K, λ(CuKα) = 1.54178 Å, Dcalc = 1.143 g/cm^3^, F(000) = 576, independent reflections 3026 [R(int) = 0.0357], 9.886° < 2θ < 145.6°, the final R1 was 0.0465 (I > 2σ(I)) and wR2 was 0.1401 (all data).

CCDC 1 446 115 and CCDC 1 446 116 contains the supplementary crystallographic data for compounds **1** and **2**. These data can be obtained free of charge via http://www.ccdc.cam.ac.uk/conts/retrieving.html (or from the CCDC, 12 Union Road, Cambridge CB2 1EZ, UK; Fax: +44 1223 336033; E-mail: deposit@ccdc.cam.ac.uk.

### 3.6. Cell Proliferation Assay

The cell proliferation of the compounds was tested using the MTT method, as reported previously [12], with 1‰ DMSO medium as the blank control. Human neuroblastoma cell line SK-N-BE (2) was maintained from American type culture collection (ATCC) and prepared in our lab. Compounds **1**–**3** were dissolved in DMSO as reserve liquid (0.1 M), which were diluted into different concentrations (2.5, 5, and 10 μM) with cell culture Medium (Eagle’s Minimum Essential Medium). SK-N-BE (2) cells (1.0 × 10^4^ cells/mL, 90 μL/well) were seeded on the 96-well plates and incubated overnight, then treated with each test compound at various concentrations (10 μL/well) at 37 °C in a humidified atmosphere containing 5% CO_2_ for 72 h. After the incubation, MTT was added to each well as described [12]. Optical density at 595 nm was measured in a 96-well microtiter plate reader (BioTek, Winooski, Vermont, USA). The optical density of formazan formed in control (untreated) cells was taken as 100% of viability. Three replicate wells were used for each control and test concentrations per microplate, and the experiment was repeated three times.

## 4. Conclusions

We isolated and characterized a new sesquiterpene, neo-acorane A (**1**), and two known ones, acoric acid (**2**) and calamusin D (**3**), from the CHCl_3_ extract of the ethanol extract of the rhizome parts of *A. calamus*. Compounds **1** and **2** possess two kinds of new chemical skeletons deriving from acorane-type sesquiterpene. The postulated biosynthetic pathways for compounds **1** and **2** deserve further confirmation by a biomimetic synthesis. Meanwhile, considering their cell proliferation activity, compounds **1**−**3** will also be potential nerve cell protection leads.

## Figures and Tables

**Table 1:** NMR spectroscopic data (500 MHz, CDCl_3_) of neo-acorane A (**1**).

Position	δ_C_, Type	δ_H_ (*J* in Hz)	HMBC (H→C)
1	94.8, C		
2	213.2, C		
3	43.4,CH_2_	a 2.63, m b 2.07, m overlap	2, 4, 5
4	34.1, CH	2.37, m	3, 5, 6, 10, 15
5	52.8, C		
6	35.4, CH_2_	a 2.43, d (17.9) b 2.24, d (17.9)	1, 4, 5, 7, 10
7	173.8, C		
8	206.9, C		
9	38.1, CH_2_	a 2.59, m b 2.50, m	5, 8, 10
10	26.1, CH_2_	a 2.11, m overlap	1, 4, 5, 6, 8, 9
		b 1.92, m	
11	30.4, CH	2.15, m overlap	1, 2, 5, 12, 13
12	18.1, CH_3_	1.02, d (7.0)	1, 11, 13
13	17.8, CH_3_	1.15, d (7.0)	1, 11, 12
14	30.4, CH_3_	2.18, s	8, 9
15	16.5, CH_3_	1.09, d (6.7)	3, 4, 5

## Supplementary Materials

The followings are available online: NMR, HRESIMS, IR, and UV spectra of compound **1** are available online at www.mdpi.com/1420-3049/22/4/529/s1, Figure S1–S9.
