# Supplementary material for: New Acorane-Type Sesquiterpene from Acorus calamus L
Source: Molecules. 2017 Mar 26;22(4):529. doi: 10.3390/molecules22040529 (PMC6153993; doi:10.3390/molecules22040529)

Supporting Information

New Acorane-Type Sesquiterpene from *Acorus calamus* L.

Juan Li [1], Jianping Zhao [2], Wei Wang [1], Lin Li [3], Lan Zhang [3], Xiao-Fang Zhao [1], Qing-Ru Liu [1], Fang Liu [1], Min Yang [4], Ikhlas A.Khan [2], Shun-Xiang Li [1,*]

Contents of Supporting Information

Figure S1 ^{1}H NMR spectrum of neo-acorane A (**1**) ($CDCl_3$, 500 MHz)

Figure S2 ^{13}C NMR spectrum of neo-acorane A (**1**) ($CDCl_3$, 125 MHz)

Figure S3 ^{1}H-^{1}H COSY spectrum of neo-acorane A(**1**) ($CDCl_3$, 500 MHz)

Figure S4 HSQC spectrum of neo-acorane A(**1**) ($CDCl_3$, 500 MHz)

Figure S5 HMBC spectrum of neo-acorane A(**1**) ($CDCl_3$, 500 MHz)

Figure S6 NOESY spectrum of neo-acorane A(**1**) ($CDCl_3$, 500 MHz)

Figure S7 IR spectrum of neo-acorane A (**1**)

Figure S8 HRESIMS of neo-acorane A (**1**)

Figure S9 UV spectrum of neo-acorane A (**1**)

Figure S1 ^{1}H NMR spectrum of neo-acorane A (**1**) ($CDCl_3$, 500 MHz)

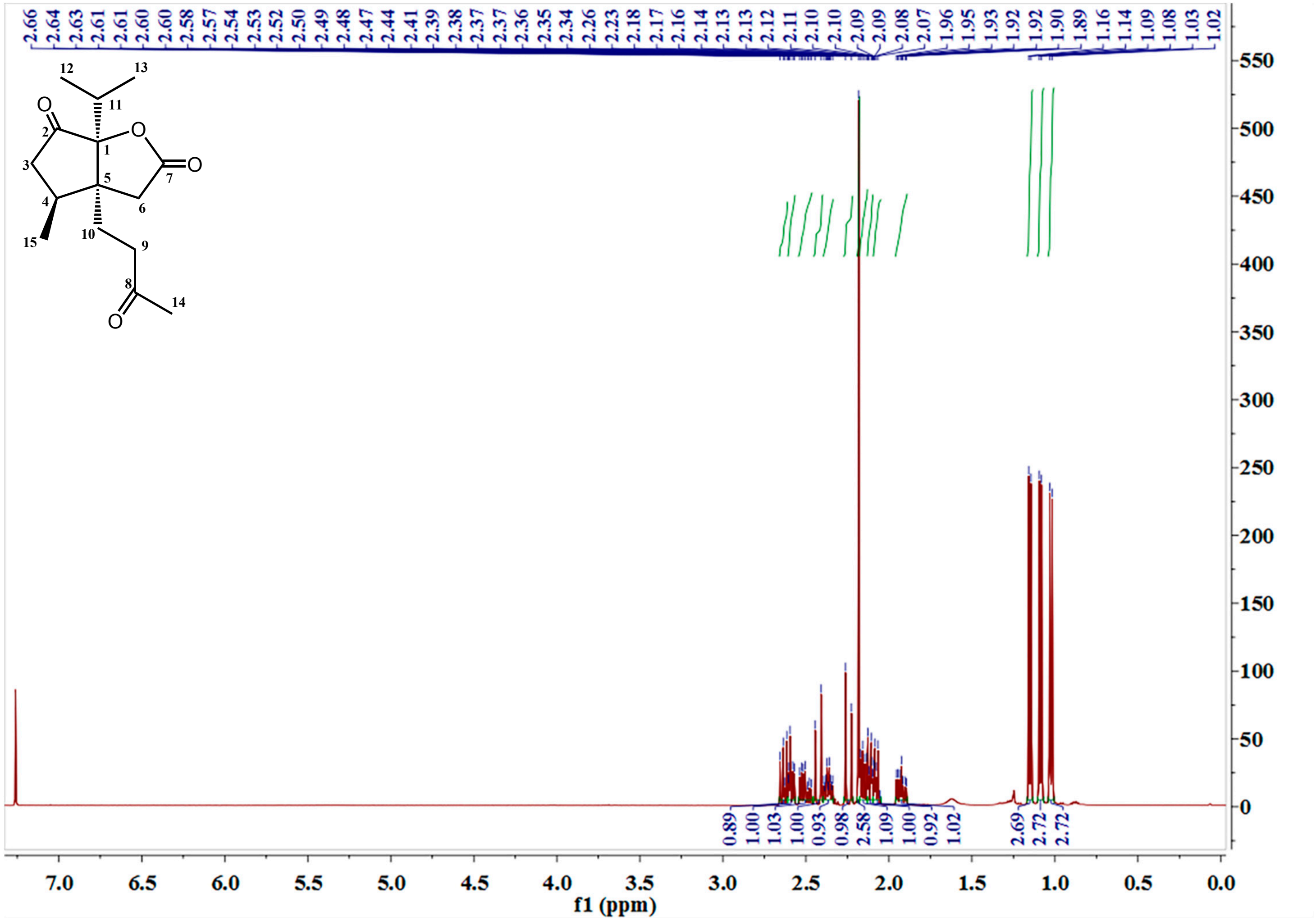

Figure S2 ^{13}C NMR spectrum of neo-acorane A (**1**) ($CDCl_3$, 125 MHz)

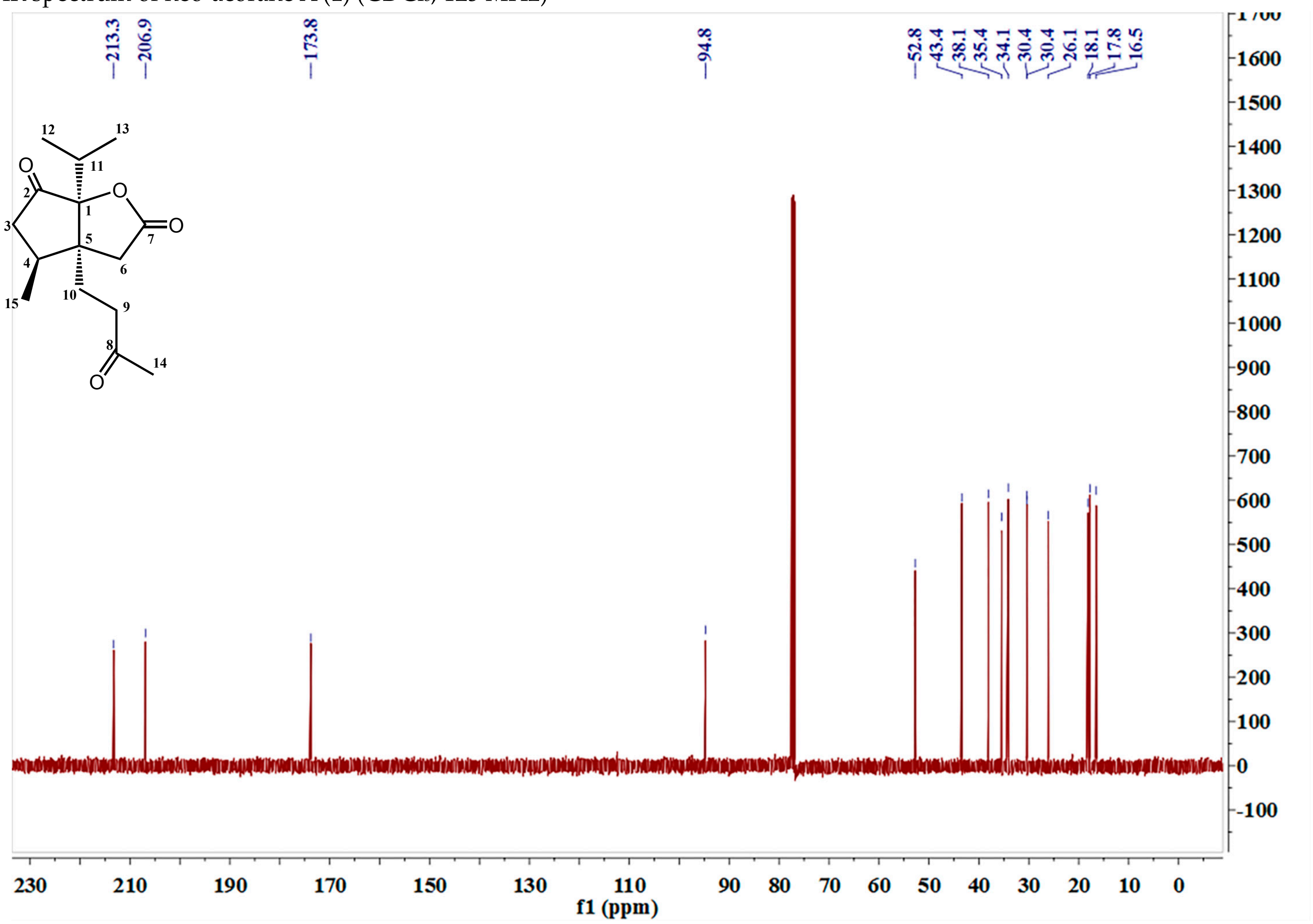

Figure S3 ^{1}H-^{1}H COSY spectrum of neo-acorane A (**1**) ($CDCl_3$, 500 MHz)

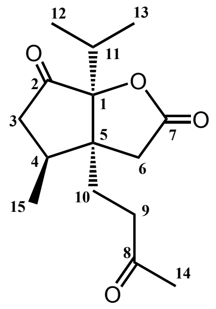

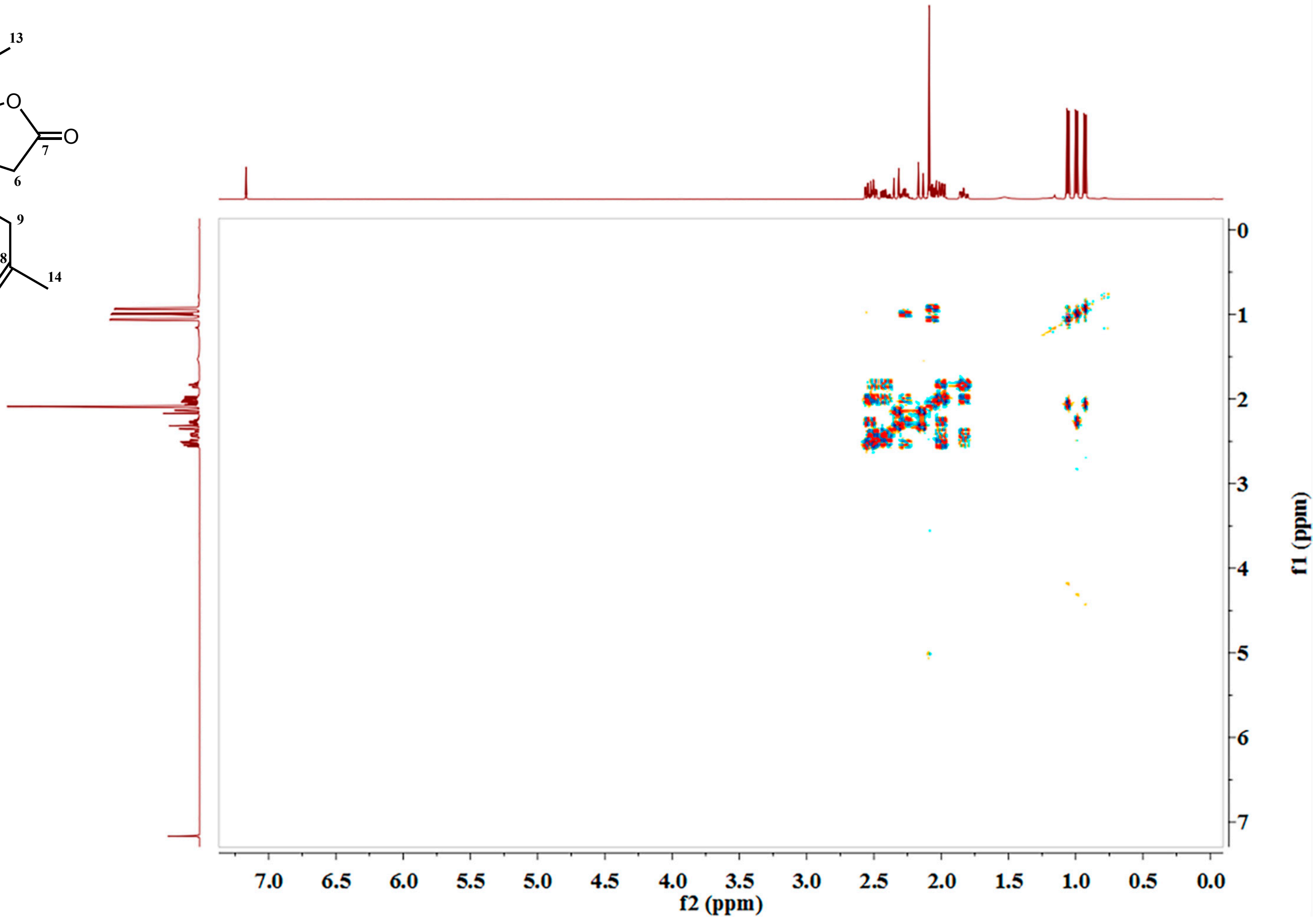

Figure S4 HSQC spectrum of neo-acorane A (**1**) ($CDCl_3$, 500 MHz)

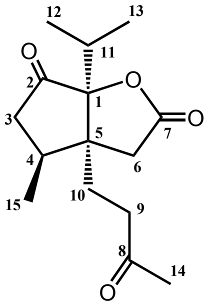

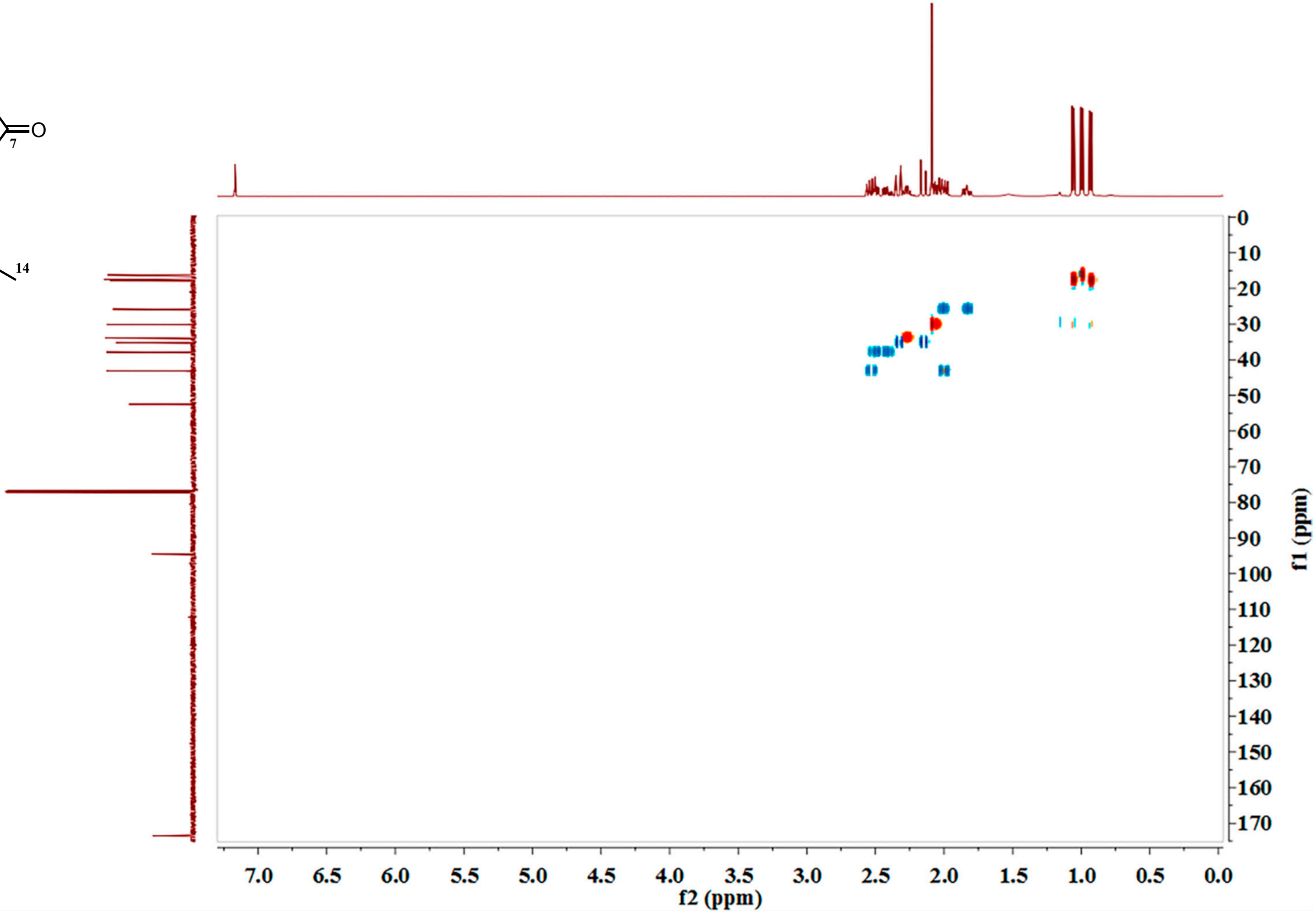

Figure S5 HMBC spectrum of neo-acorane A (**1**) ($CDCl_3$, 500 MHz)

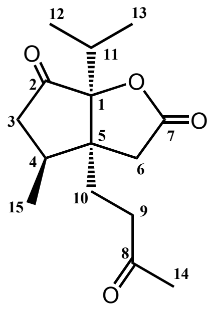

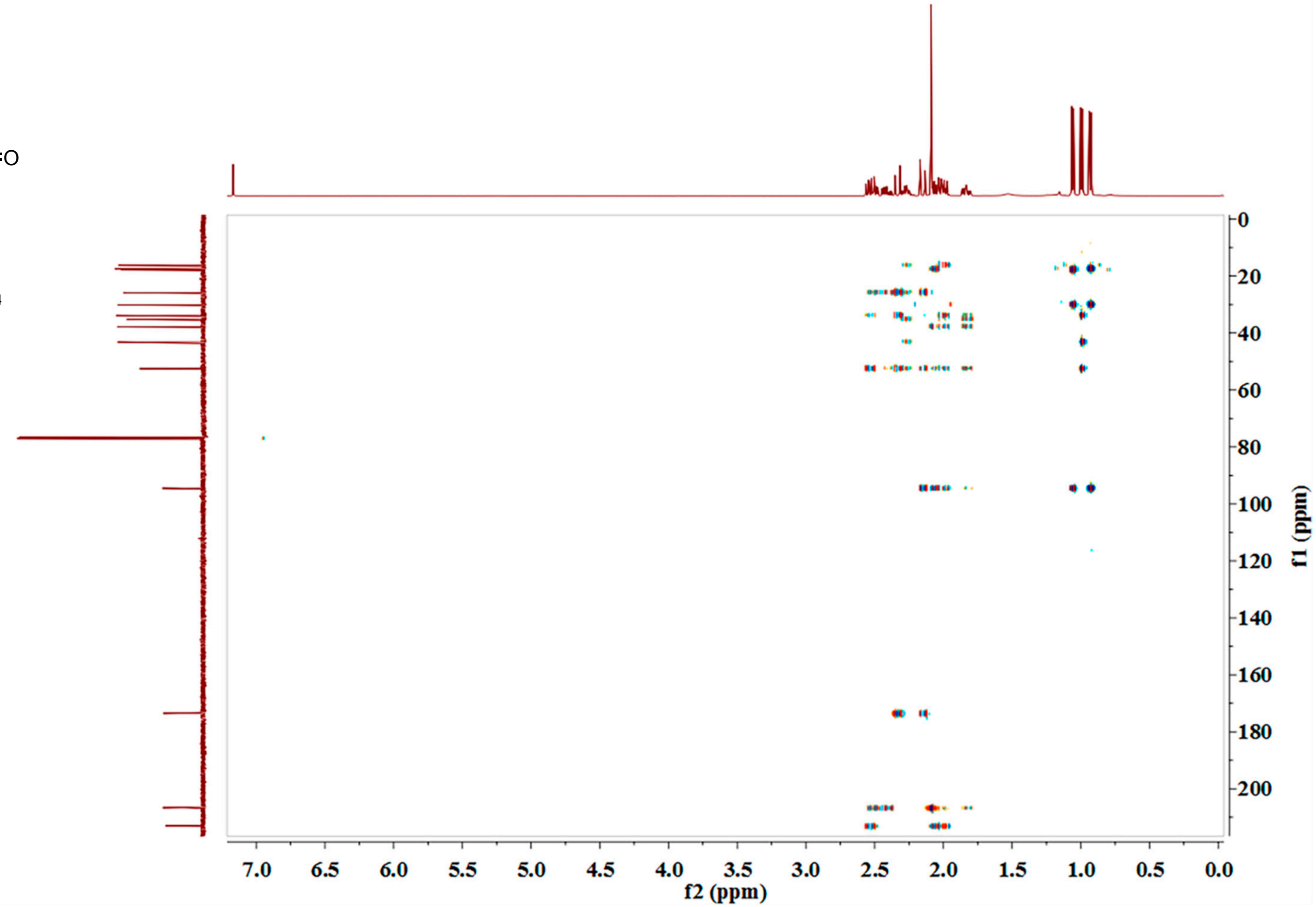

Figure S6 NOESY spectrum of neo-acorane A (**1**) ($CDCl_3$, 500 MHz)

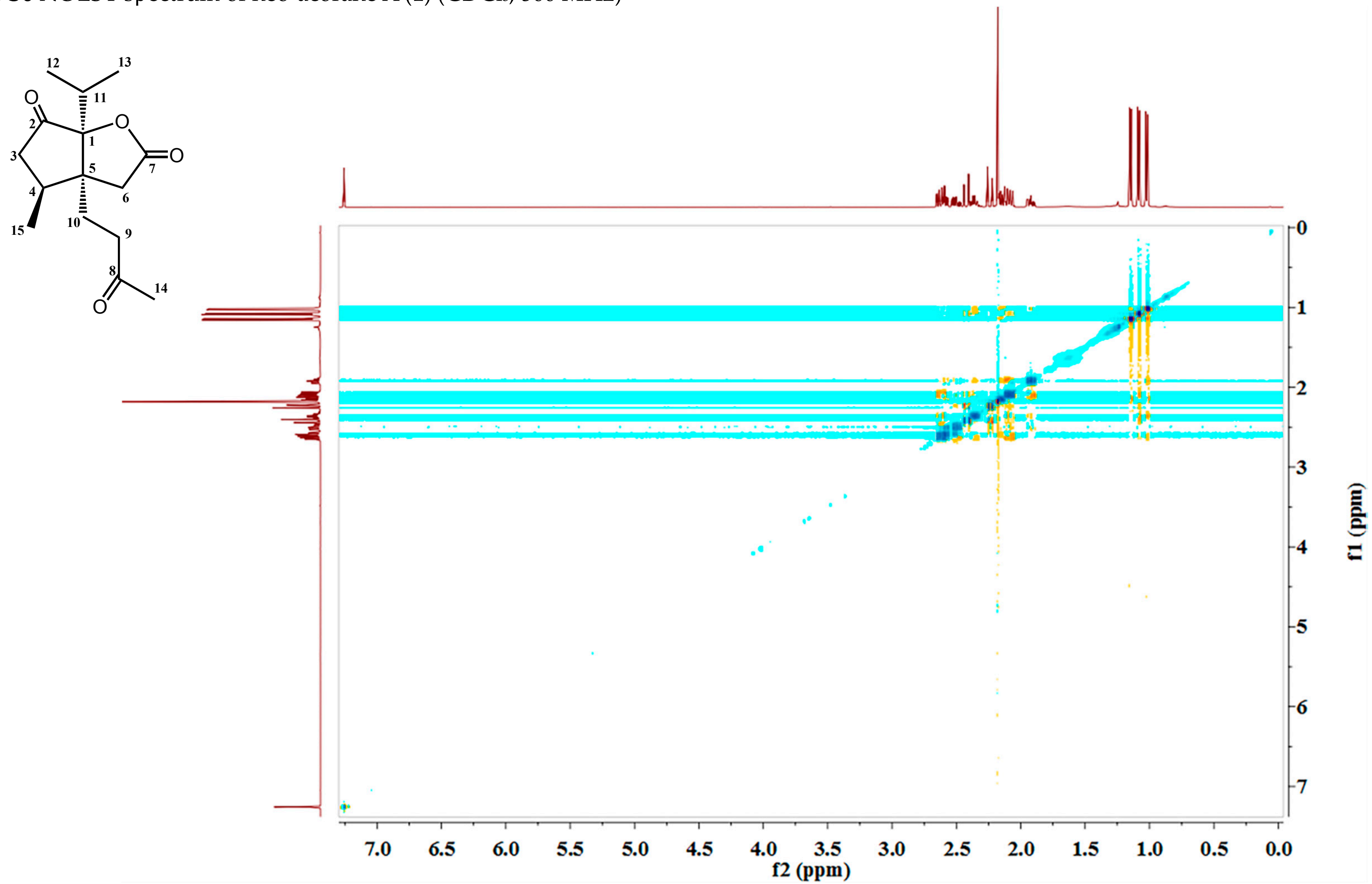

Figure S7 IR spectrum of neo-acorane A (**1**)

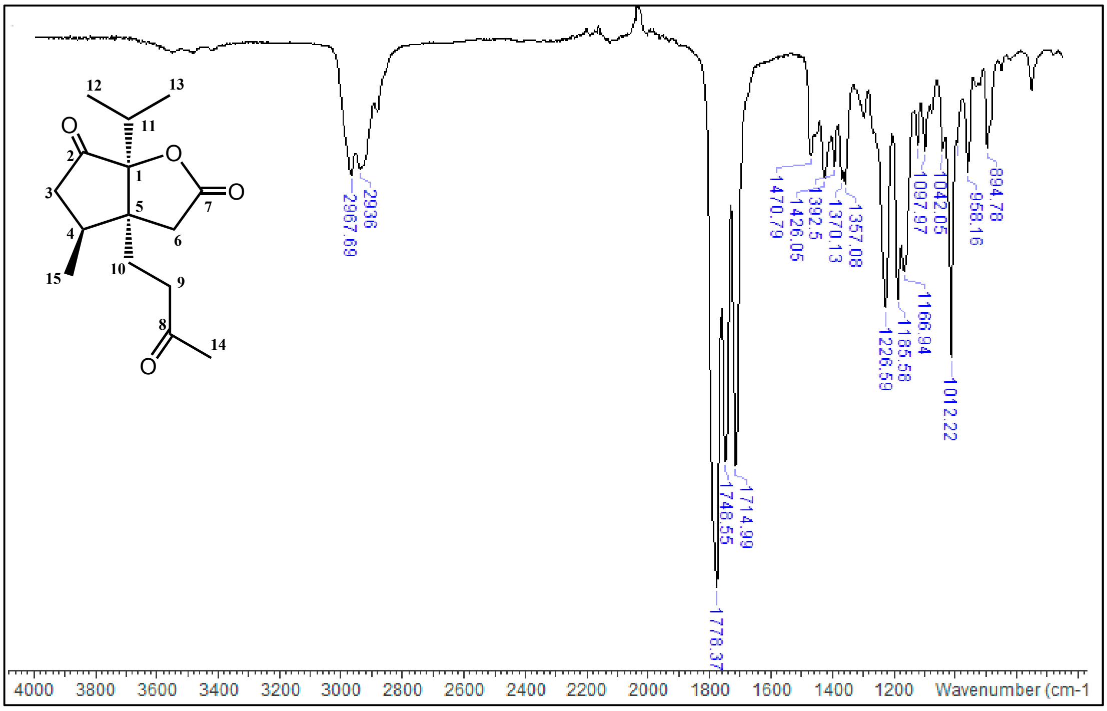

Figure S8 HRESIMS of neo-acorane A (**1**)

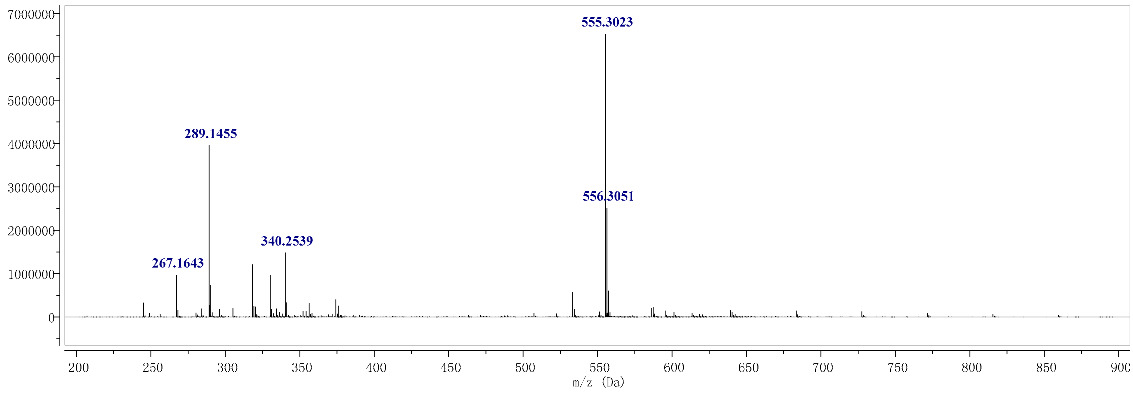

Figure S9 UV spectrum of neo-acorane A (**1**)

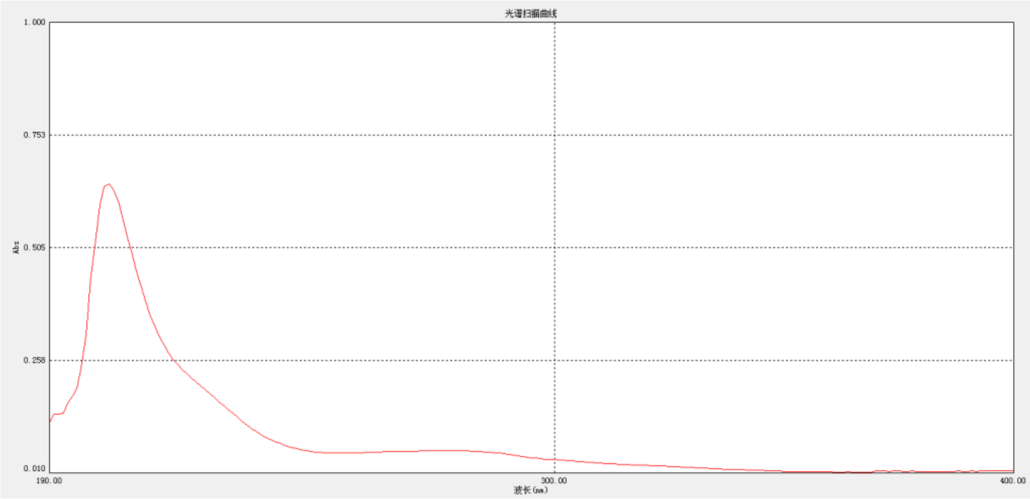

Supplement: Supplementary file 1 [file molecules-22-00529-s001.pdf]
